# Pitfalls of Hypoglycemia: Uncommon Presentation of Hirata’s Disease in a Caucasian Female

**DOI:** 10.7759/cureus.77671

**Published:** 2025-01-19

**Authors:** Dinara Salimova, Tatevik Aloyan, Mohammed Haroun

**Affiliations:** 1 Internal Medicine, Ascension Saint Joseph, Chicago, USA; 2 Internal medicine, Ascension Saint Joseph, Chicago, USA

**Keywords:** autoimmune hypoglycemia, hirata's disease, hypoglycemia workup, insulin autoimmune syndrome, whipple's triad

## Abstract

Insulin autoimmune syndrome (IAS) is a known cause of hypoglycemia, more commonly observed in Asian populations, but it is rarely seen in Western countries. We present a case of recurrent hypoglycemia in a 53-year-old Caucasian female, attributed to insulin autoantibodies, which responded well to treatment with steroids and dietary modifications. Increasing awareness of IAS among clinicians is crucial to prevent unnecessary, costly, and potentially harmful diagnostic procedures when investigating recurrent and refractory hypoglycemia, and to ensure appropriate management strategies are implemented.

## Introduction

Insulin autoimmune syndrome (IAS), also known as Hirata's disease, is a rare hypoglycemic disorder first described in 1970 in Japan by Hirata and colleagues. Nowadays, the condition is characterized as an autoimmune disease manifesting with recurrent hypoglycemia in association with elevated levels of serum insulin and the presence of insulin autoantibodies. It was initially defined as a condition that develops without prior exposure to exogenous insulin; however, recent data also suggest that it can be seen in individuals previously exposed to it [1,2].

According to the statistics, Hirata’s disease is mainly seen in individuals over 40 and occurs equally in both females and males [3,4]. Since the condition was first identified, the majority of cases have been reported in Japan and are generally more prevalent in Asian populations compared to Western populations. As a consequence of the rarity of the disease and poor awareness, many cases remain misdiagnosed or identified late which leads to inappropriate management and unnecessary workup [5]. Moreover, some clinical occasions, such as sepsis, alcohol intoxication, malnutrition, insulin overdose, or drug overdose, may present with hypoglycemia and the condition could be misinterpreted. Rising awareness about the IAS among clinicians is important for prompt diagnosis and application of effective treatment strategies. We present a case of Hirata’s disease in a 53-year-old Caucasian female not previously exposed to insulin, that manifested as recurrent hypoglycemia.

## Case presentation

A 53-year-old Caucasian female with a medical history significant for hyperlipidemia and hypertension presented to the emergency department with three weeks of nausea, vomiting, and dizziness. She had several episodes of nonbilious non-bloody vomiting containing food particles. During this period, her oral intake has significantly reduced, and she has lost approximately 20 pounds. She denied chest pain, shortness of breath, abdominal pain, fevers or chills. On further questioning, she reported that during the last two years, she has had episodic recurrent anxiety, diaphoresis, weakness, and palpitations that happened one to three hours after meals or in the morning before breakfast and used to improve after intake of sweets or snacks. During those past two years, she was also told by her primary care physician that her glucose values have been on the lower side; however, she does not recall exact levels and states that it did not necessitate further evaluation. She denied any change in her urinary or bowel habits. The list of medications included amlodipine 5 mg daily and atorvastatin 20 mg daily. She denied the use of any new medications, dietary supplements, illicit drugs, or alcohol. She was admitted to the Internal Medicine unit for further management.

**Figure 1:**
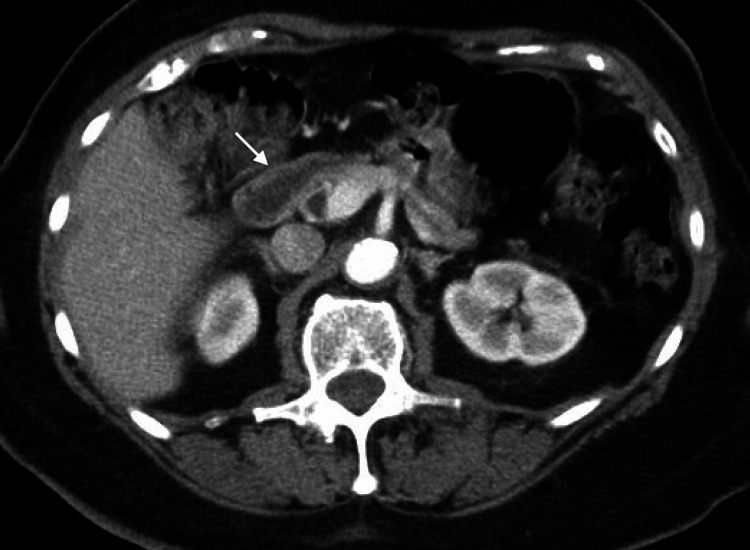
CT of the abdomen and pelvis with IV contrast The white arrow indicates the pancreas, no lesions resembling insulinoma were identified. IV - intravenous

Upon presentation, the patient was feeling nauseous and complained of abdominal discomfort. She was hemodynamically stable, nonfebrile, and saturating well on room air. Physical examination was remarkable only for mild discomfort with palpation in the epigastric area. Body mass index was 26 kg/m^2^. Complete blood count and comprehensive metabolic panel values were normal except for glucose of 45 mg/dL. The pregnancy test, blood alcohol level, and urine toxicology screen were negative. Computed tomography (CT) of the abdomen and pelvis with intravenous (IV) contrast was unremarkable, showing normal pancreas and gastrointestinal tract, and no lesions resembling insulinoma (Figure 1). The results of laboratory investigations are summarized in Table 1.

**Table 1:** Summary of laboratory workup

	Values	Units	Normal range
White blood cell count	11.2	k/mm cu	4.0 - 11.0
Platelets	294	k/mm cu	150 - 450
Hemoglobin	14.9	g/dL	13.0 - 17.0
Glucose, blood	40 - 45	mg/dL	70 - 99
Total protein	7.5	g/dL	6.4 - 8.9
Albumin	4.5	g/dL	3.5 - 5.7
Serum creatinine	0.67	mg/dL	0.7 - 1.3
Glomerular filtration rate (GFR)	> 60	mL/min/1.73m^2^	> 60
Aspartate aminotransferase (AST)	30	IU/L	13 - 39
Alanine aminotransferase (ALT)	25	IU/L	7.0 - 52.0
Alkaline phosphatase	69	IU/L	35 - 104
Total bilirubin	0.7	mg/dL	0.3 - 1.0
Lipase	9	IU/L	11 - 82
Blood alcohol level	< 10	mg/dL	0 - 10
Thyroid-stimulating hormone	1.796	µIU/mL	0.27 - 4.20
Morning cortisol level	15.3	mcg/dL	6.2 - 19.4

She was started with the hypoglycemia protocol and maintenance fluid with 5% dextrose in lactated ringer solution (D5-LR) with glucose values maintaining mostly in the 70-80s. She has also received intravenous ondansetron for the management of nausea. While the right upper quadrant ultrasound did not reveal signs of cholecystitis or bile duct dilatation, the hepatobiliary imidoacetic (HIDA) scan was suggestive of possible cholecystitis and the patient underwent laparoscopic cholecystectomy. Pathology confirmed acute cholecystitis. Symptoms of nausea, vomiting, and abdominal discomfort resolved afterward with the return of appetite and ability to tolerate food. Surprisingly, despite well-tolerated regular scheduled meals every three to four hours, the patient still developed neuroglycopenic symptoms one to two hours after food intake while her blood glucose levels were 40s. She remained on 5% dextrose in lactated ringer solution infusion.

The patient underwent a supervised fasting test. After two hours of fasting, she developed symptomatic hypoglycemia with a blood glucose level of 44 mg/dL, which resolved shortly after oral glucose administration. Her insulin level was elevated at 118 mU/mL (normal range: ≤18.4 µIU/mL), and her C-peptide level was 3.5 ng/mL (normal range 0.80-3.85 ng/mL). Endocrinology was consulted and the patient underwent workup for hypoglycemia. Urinalysis was unremarkable and negative for sulfonamides. The anti-insulin antibody level was 118 U/mL (normal range: <0.4 U/mL). She had an HbA1c of 5.5% (Table 2).

**Table 2:** Summary of laboratory workup

Test	Result	Units	Normal Range
Insulin (2 hours after fasting)	118	uIU/mL	≤18.4
C-peptide (2 hours after fasting)	3.5	ng/mL	0.80-3.85
Insulin autoantibodies	108	U/mL	< 0.4
HbA1c	5.5	%	<5.7%

Noteworthy, the patient did not have a history of gastric bypass surgery in the past and there was no family history of hypoglycemia, personal history of diabetes, or exposure to insulin products. The patient denied a history of either autoimmune or hematologic disorders or diabetes. He had a negative hepatitis panel and an anti-nuclear antibody panel.

Based on the episodes of hypoglycemia, high levels of insulin, high-normal levels of C-peptide, and positive insulin autoantibodies, the patient was diagnosed with IAS. She was started on low carbohydrate meals and oral prednisone 60 mg per day, with slow tapering over three months. The patient responded well to the treatment with the resolution of hypoglycemia. Upon follow-up six months after her initial presentation, she declined repeated neuroglycopenic symptoms and was normoglycemic.

## Discussion

It is hypothesized that predisposition to IAS is determined by an individual's ethnic and genetic background. HLA plays a major role in the development of Hirata’s disease with HLA-DRB1∗0406 being more frequently seen in the East Asian population, particularly in Japanese, and HLA-DRBQ *0403 occurring in affected Caucasians, which likely explains the variety of distribution of cases among different ethnic groups [2,5]. There also has been an observation that more recently the incidence of the disease has been rising more evenly worldwide [4]. Nearly 80% of cases were associated with other autoimmune disorders, such as systemic lupus erythematosus, rheumatoid arthritis, and others. Some cases were also described among individuals with hematologic diseases, including multiple myeloma or benign monoclonal gammopathy [3]. 

Although the exact etiology of IAS is still unclear, it was shown that it can be triggered by some viruses, such as coxsackie B, influenza, mumps, rubella, and hepatitis C; as well as medications, particularly sulfur/sulfhydryl groups containing ones, among which methimazole is the most commonly seen [2,6,7]. However spontaneous cases were also described [5]. 

Hirata’s disease is considered to represent a novel type VII hypersensitivity, which is characterized by the presence of autoantibodies attacking circulating target proteins or hormones [8]. Endogenous autoantibodies, namely IgG, IgA, and IgM, with IgG being the most common, bind insulin/proinsulin, resulting in loss of their ability to reduce blood glucose levels. During the postprandial state rising blood glucose levels stimulate the secretion of insulin products which become bound by insulin autoantibodies. As a result, patients become prone to postprandial temporary hyperglycemia that promotes continuous insulin production by the pancreatic beta-cells which ceases only when unbound active insulin rises to the level when endogenous antibodies binding capacity is exceeded [3,9]. Thus, insulin/proinsulin bound to endogenous antibodies in blood serves as a reservoir with a variable dissociation rate. These complexes become dissociated after the levels of unbound insulin start to drop, either in the postprandial or fasting state, leading to the release of more active insulin into the blood and hypoglycemia [10]. Additionally, sustained levels of insulin suppress the effects of counterregulatory hormones. The mechanisms mentioned above result in overall abnormal glucose metabolism with phases of hyper and hypoglycemia.

Clinical presentation varies from mild and transient to prolonged and severe life-threatening hypoglycemic episodes requiring plasmapheresis. Alternating episodes of hypoglycemia and hyperglycemia can also be observed, as described above [2]. The severity, duration, and swing from hyper- to hypoglycemia have been attributed to endogenous antibody characteristics, particularly their affinity, titers, and capacity [11].

Differential diagnoses should include insulinoma, extrapancreatic neoplasms, use of sulfonamides, and exogenous insulin administration. In Japan, IAS is the third leading cause of hypoglycemia after insulinoma and extrapancreatic neoplasms, which makes IAS an important diagnosis that should be considered among differentials [2]. The first step in the diagnosis of Hirata’s disease should be confirmation of hypoglycemia. According to the Endocrine Society Clinical Practice Guideline, the patient should undergo further workup for hypoglycemia if it is confirmed by the presence of Whipple’s triad (symptoms of hypoglycemia in the setting of low glucose level, at least below 55 mg/dL, and resolution of symptoms after glucose intake) [12]. The second step is the determination of insulin and C-peptide levels. Hirata’s disease is characterized by an increased level of insulin and usually a normal level of C -peptide. This phenomenon is explained by the fact that insulin develops a longer half-life due to the presence of insulin-antibody complexes that serve as a reservoir, however, as there are generally no C-peptide-antibody complexes, the half-life of this marker remains unaffected. On the other hand, if antibodies can bind C-peptide and proinsulin, those products will have an increased half-life resulting in raised levels on laboratory assays. Therefore, C-peptide analysis cannot reliably distinguish between autoimmune and factitious hypoglycemia [13,14].

Besides withdrawal from the triggering factors, the mainstay of management of IAS is dietary modifications with the prevention of rapid rise in glucose after meals and avoidance of long fasting periods. Meals should be frequent and small, low in carbohydrates [3]. It is recommended to take cornstarch, a glucose polymer characterized by the slow rate of intestinal absorption. In more severe cases, alpha-glucosidase inhibitors, such as acarbose and voglibose can be recommended to delay carbohydrate absorption. The disease is generally considered to be self-limiting with a tendency to regress in three to six months after withdrawal of the triggering substance. However, refractory cases have also been described and have been successfully treated with steroids, plasmapheresis, rituximab, azathioprine, and other immunosuppressants [2,4,7].

Our 53-year-old Caucasian female experienced both postprandial and fasting hypoglycemia, accompanied by elevated insulin levels and the presence of insulin autoantibodies. The hypoglycemic episodes resolved after three months of treatment with prednisone and did not recur. Based on her history of hypoglycemic symptoms and previous indications of low blood sugar, it is hypothesized that her case of IAS is spontaneous, though it may have been exacerbated by acute cholecystitis. Insulinoma was ruled out through a negative CT scan of the abdomen and pelvis and a normal C-peptide level. Urinalysis was negative for sulfonamide use. The normal (non-suppressed) C-peptide level during episodes of spontaneous hypoglycemia further excludes the possibility of factitious hypoglycemia.

## Conclusions

IAS is a well-recognized cause of hypoglycemia in the Asian population, though it remains uncommon in Western populations. Rising awareness of this condition is essential to prevent unnecessary, prolonged, and costly diagnostic procedures aimed at identifying the cause of hypoglycemia. Knowledge of the underlying mechanisms of hypoglycemia in Hirata’s disease is important for accurate interpretation of laboratory findings while understanding its pathogenesis is critical for effective management.
